# Analysis of Postoperative Knee Dysfunction Following a Distal Femur Fracture Managed by Different Surgical Methods

**DOI:** 10.7759/cureus.109955

**Published:** 2026-05-31

**Authors:** Swapnali D Jadhav, Sandeep Shinde, Siddhi P Patrekar, Sawani Aphale

**Affiliations:** 1 Department of Musculoskeletal Sciences, Krishna College of Physiotherapy, Krishna Vishwa Vidyapeeth (Deemed to be University), Karad, IND

**Keywords:** distal femur fracture, gait abnormality, knee dysfunction, limb length discrepancy, supracondylar femur fracture

## Abstract

Background

Distal femur fractures, in particular, are complicated injuries that have a major effect on knee biomechanics and functionality. As all fractures primarily affect older females from low-energy falls and younger males from high-energy injuries, patients treated with open reduction-internal fixation (ORIF) and intramedullary nailing (IMN) had their postoperative knee dysfunction and functional results assessed in this study.

Objective

This study aimed to analyze postoperative knee dysfunction and functional outcomes following distal femur fracture managed by ORIF and IMN and to evaluate functional outcomes, range of motion (ROM), muscle strength, and functional recovery.

Methods

A cross-sectional study was conducted with 108 patients (54 ORIF, 54 IMN) who had distal femur fractures and were at least 18 years old. Demographic data, injury details, and surgical approaches were recorded. Outcomes included ROM, muscle strength, limb length, and girth measurements. Complications like stiffness, shortening, extensor lag, and functional outcomes (Schatzker and Lambert criteria) were assessed.

Result

Multiple dysfunctions were identified despite successful surgical fixation. Knee stiffness was the most frequent limitation, followed by quadriceps wasting and gait deviations. Extensor lag and limb shortening were also noted in a significant subset. Gait abnormalities were observed in some patients, most commonly antalgic gait and knee flexion avoidance gait. Functional outcome assessment revealed a significantly higher rate of excellent results in the IMN group. ORIF has been associated with increased rates of infection and knee stiffness.

Conclusion

This study highlights that postoperative knee dysfunction, including stiffness, quadriceps weakness, extensor lag, and gait deviations, is common following distal femur fracture surgeries, regardless of fixation type. Early mobilization and proper fixation techniques are crucial for recovery. IMN provides better functional outcomes and fewer complications compared to ORIF for supracondylar femur fractures.

## Introduction

A fracture of the supracondylar femur involves the distal portion of the femur, specifically the metaphyseal region, encompassing the distal 8-15 cm. These fractures often extend into the articular surface, leading to significant joint involvement and making management challenging [1]. Although distal femur fractures are relatively uncommon compared to other long bone fractures, they are considered severe due to their complex anatomy and the functional importance of the knee joint [2]. Distal femur fractures are commonly classified using the AO/Orthopaedic Trauma Association (OTA) system, which categorizes them into type A (extra-articular), type B (partial articular), and type C (intra-articular) fractures, with further subdivisions based on comminution and fracture pattern [3]. Epidemiological data indicate that distal femur fractures constitute approximately 3-6% of all femoral fractures and about 0.4% of all fractures. A bimodal distribution is evident: young male patients often sustain high-energy injuries, such as road traffic accidents, while older female patients are more likely to experience low-energy mechanisms, typically falls from standing height, often associated with osteoporosis [4]. Major etiological factors include motor vehicle collisions, falls from height, and direct trauma [5]. Despite this progress, these fractures remain difficult to treat because of complicating factors such as osteoporosis, severe comminution, intra-articular extension, open wounds, and associated soft tissue injuries involving the ligaments, menisci, and extensor mechanism [6].

Nevertheless, comminuted fractures, poor bone quality, and inadequate fixation can lead to adverse outcomes such as malalignment, implant failure, infection, and delayed union [7]. Restoration of length, alignment, and articular congruity, along with early mobilization, is central to successful management [8]. Operative fixation is usually achieved with intramedullary nailing or plate osteosynthesis, each with specific advantages and limitations [7]. Stable fixation is critical for promoting bone healing, maintaining biomechanical stability [9], and allowing early physiotherapy interventions that prevent long-term disability. Even after successful union, many patients experience postoperative knee dysfunction, manifested as reduced range of motion (ROM), extensor lag, quadriceps weakness, persistent pain, impaired gait, and an increased risk of post-traumatic osteoarthritis [10]. The proximity of the fracture to the knee joint makes achieving full functional recovery especially difficult, as both joint congruity and soft tissue balance play crucial roles in mobility and long-term outcomes [11]. Historically, conservative management with traction and prolonged immobilization was employed; however, this approach was often associated with complications such as malunion, nonunion, joint stiffness, and muscle atrophy. With advances in surgical techniques, operative management has become the mainstay, providing superior outcomes in terms of stability, alignment, and functional recovery [6].

Physiotherapy is essential in the recovery process, aiming to restore ROM, muscle strength, and overall functional independence. Early, structured, and criteria-based rehabilitation programs have been shown to reduce complications such as stiffness, contractures, and muscle wasting, ultimately improving patients' quality of life [12]. However, standardized physiotherapy protocols specific to supracondylar femur fractures remain lacking. Most available evidence consists of isolated case reports and small series, often with inconsistent methodologies, which limits their generalizability [13]. The global burden of distal femur fractures is significant, not only because of the functional impairment they cause but also due to their socioeconomic impact. Prolonged hospitalization, high surgical costs, delayed return to work, and the need for long-term rehabilitation contribute to a considerable healthcare challenge, particularly in resource-limited settings [14]. Flexion deficits are particularly problematic in populations where kneeling and squatting are essential for daily activities, such as in many Asian and Middle Eastern communities [15].

Given these challenges, it is imperative to evaluate how different surgical methods influence postoperative function and rehabilitation outcomes. While both open reduction-internal fixation (ORIF) and intramedullary nailing (IMN) provide effective stabilization, their impact on functional recovery, particularly in relation to physiotherapy outcomes, remains underexplored. The current study, therefore, aims to address this gap by comparing postoperative knee dysfunction between patients treated with ORIF and those treated with IMN. By analyzing parameters such as knee ROM, extensor lag, quadriceps strength, gait quality, pain, and time to independent ambulation, this study seeks to provide evidence-based insights that will support physiotherapists in tailoring rehabilitation strategies. Ultimately, the findings may contribute to the development of more structured rehabilitation protocols, enhancing long-term outcomes and quality of life for patients with distal femur fractures.

## Materials and methods

The present cross-sectional study was conducted at Krishna Vishwa Vidyapeeth (Deemed to be University), Karad, India, with the purpose of evaluating postoperative knee dysfunction in patients who sustained supracondylar femur fractures. A total of 108 patients were enrolled through simple random sampling.

All participants presented with distal femur fractures and were surgically managed either by ORIF using a locking compression plate or by IMN. Based on the method of fixation, participants were divided into two groups: Group A, consisting of patients treated with ORIF, and Group B, comprising those who underwent IMN.

Fractures were further categorized according to the AO/OTA classification system for distal femur fractures to ensure standardized fracture assessment and comparison between groups. Type 33-A represented extra-articular fractures, type 33-B represented partial articular fractures, and type 33-C represented complete articular fractures. This classification was used to document fracture severity and pattern distribution in both the ORIF and IMN groups.

Inclusion criteria included patients between 18 and 55 years of age, both genders (male and female), normal BMI, those fractures managed surgically with according to the AO/OTA classification (type 33-A (extra-articular) distal femur fractures were primarily managed with IMN, whereas type 33-B (partial articular) and type 33-C (complete articular) fractures were managed with ORIF), those who provided informed consent, and those available for a minimum of one year of postoperative follow-up. Exclusion criteria included pathological fractures, periprosthetic supracondylar fractures, patients with pre-existing knee pathology such as severe osteoarthritis or previous ligament or meniscus surgery, individuals outside the specified age range, and those unwilling to participate.

Mechanism of injury was classified as high-energy trauma, including road traffic accidents, falls from height, and major direct trauma, and low-energy trauma, defined as falls from standing height or minor household trauma resulting in fracture.

Ethical approval was obtained from the Institutional Ethics Committee of Krishna Vishwa Vidyapeeth (Deemed to be University) (approval number: KVV/IEC/09/2023). Each participant was approached personally and informed about the study objectives, procedures, and potential implications before obtaining verbal consent. Demographic details, mode of injury, and specifics of the surgical approach were meticulously documented. Postoperative evaluation involved measuring the ROM of the knee with a goniometer and evaluating limb length and thigh girth.

**Figure 1:**
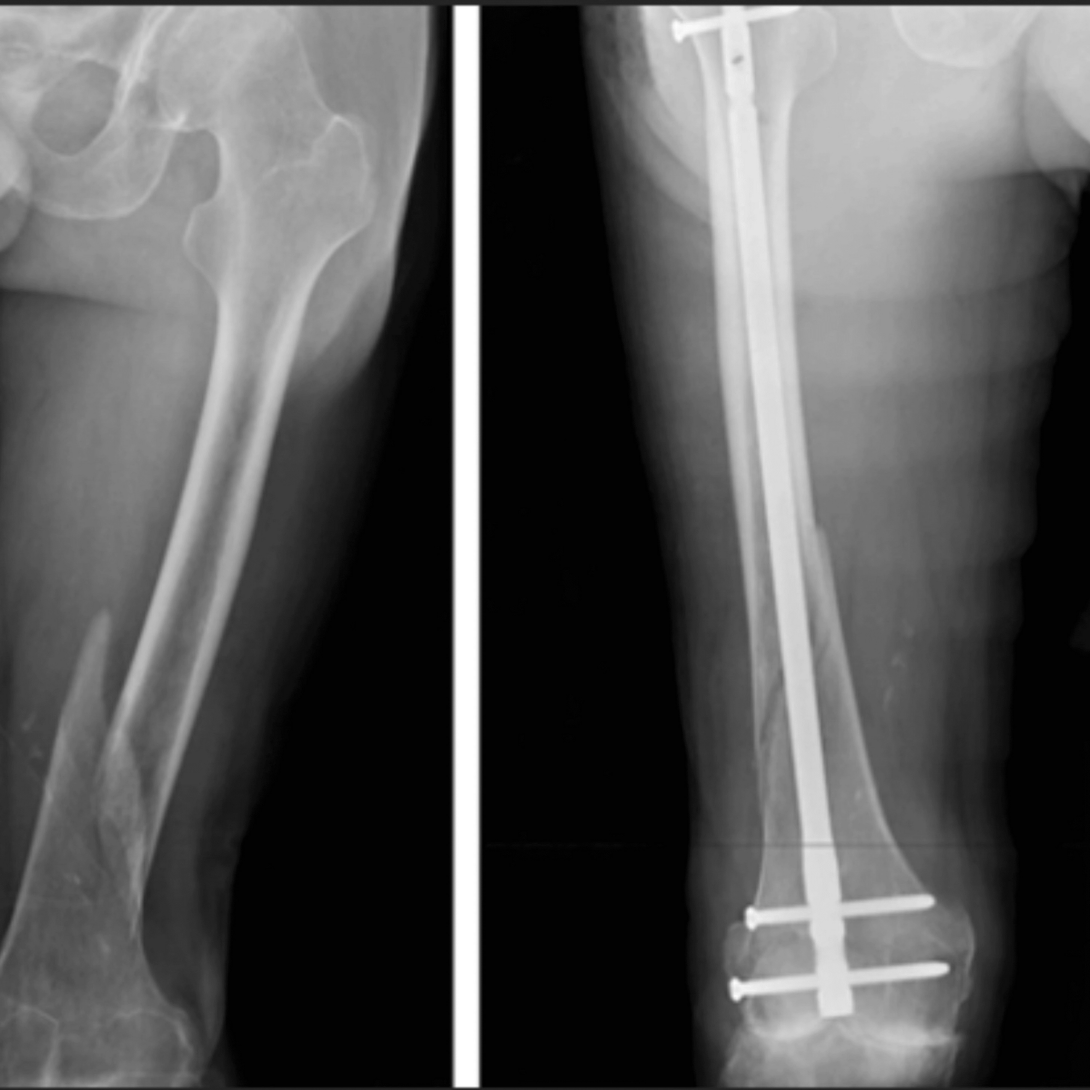
Type 33-A (extra-articular) distal femur fracture and intramedullary nailing with screws

**Figure 2:**
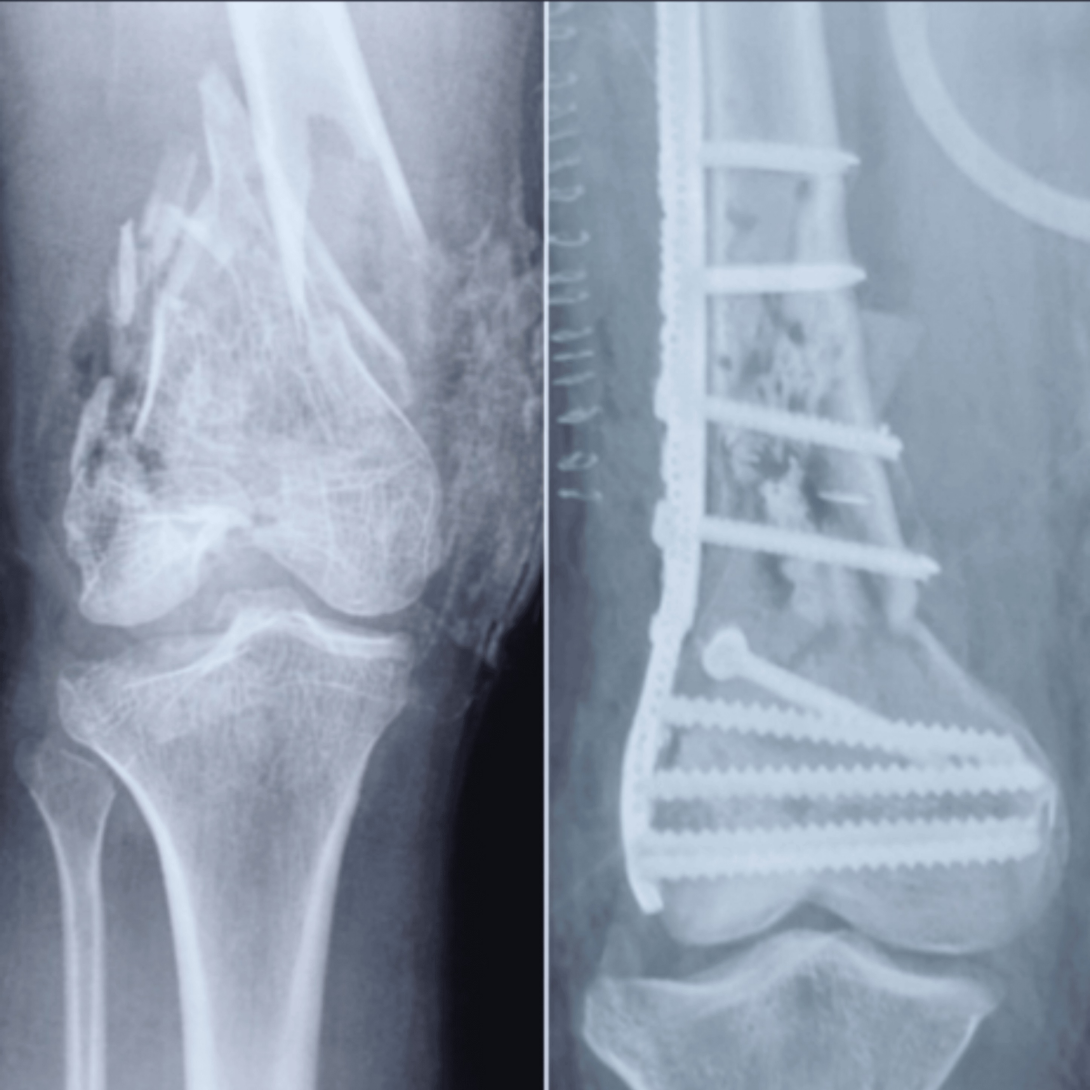
Type 33-C compound comminuted right distal femur fracture and open reduction-internal fixation with plating

These parameters were recorded at a minimum of one-year follow-up to compare functional outcomes and residual knee dysfunction between the two surgical groups, as given in Figure 1 and Figure 2.

Outcome measures

ROM

The knee joint's flexion and extension were assessed using a goniometer. It was explained to the individual to move the joint through its ROM immediately after the goniometer was set to zero. Normal knee flexion is 0-135 degrees.

Manual Muscle Testing

Muscle strength of the knee extensors (quadriceps) and knee flexors (hamstrings) was assessed using standard Manual Muscle Testing principles based on the Medical Research Council (MRC) scale grading system (0-5). Knee extensors were assessed in a sitting position, and knee flexors were assessed in a prone or sitting position, depending on patient comfort and available range. In patients with restricted knee ROM, muscle strength was assessed within the available pain-free range using a modified break test approach to ensure consistency and clinical applicability [16].

Limb Length

Leg length was assessed by measuring the distance between the anterior iliac spine and the tip of the medial malleolus. The length of the lower limb was compared.

Girth Measurement

Lower limb girth measurements are frequently performed to assess muscle mass or atrophy, particularly in people with musculoskeletal disorders.

Gait Analysis

Gait pattern was clinically assessed during walking observation to identify postoperative gait deviations, including antalgic gait, knee flexion avoidance gait, quadriceps avoidance gait, and reduced knee flexion during swing phase.

Functional Outcome Assessment

Overall functional outcome was assessed using the Schatzker and Lambert criteria, which classify outcomes as excellent, good, fair, or poor based on pain, ROM, deformity, and functional ability.

Statistical analysis

IBM SPSS Statistics for Windows, Version 26.0 (IBM Corp., Armonk, New York, United States), was used to analyze the data. Descriptive statistics, including mean, standard deviation, and percentages, were used to generate the clinical and demographic data. The chi-squared test was utilized for categorical data and the independent t-test for continuous variables. Statistical significance was defined as a p-value of less than 0.05.

## Results

The study has 108 individuals divided evenly into two groups: 54 had ORIF treatment and 54 had IMN.

Table 1 shows the study population's demographic information, contrasting patients who received IMN with those who received ORIF. In both ORIF and IMN, patients were most common in the 18-30-year age group (ORIF: 59.3%; IMN: 64.8%), followed by those in the 31-40-year (ORIF: 27.8%; IMN: 25.9%) and 40-55-year age groups (ORIF: 12.9%; IMN: 9.2%). There was no statistically significant difference in the two groups' age distributions (p=0.908).

**Table 1:** Demographic Variables ORIF: open reduction-internal fixation; IMN: intramedullary nailing; OTA: Orthopaedic Trauma Association

	Group A (ORIF) (n=54)	Group B (IMN) (n=54)	P-value
Age group (years)			
18-30	32 (59.3%)	35 (64.8%)	0.908
31-40	15 (27.8%)	14 (25.9%)	
40-55	7 (12.9%)	5 (9.2%)	
Gender			
Male	43 (79.6%)	38 (70.4%)	0.03
Female	11 (20.4%)	16 (29.6%)	
Surgical approach			
High-energy trauma	41 (75.9%)	45 (83.3%)	0.25
Low-energy trauma	13 (24.1%)	9 (16.7%)	
AO/OTA classification			
33-A	12 (22.2%)	38 (70.4%)	0.001
33-B	14 (25.9%)	10 (18.5%)	
33-C	28 (51.9%)	6 (11.1%)	

Regarding the distribution by gender, in both groups, men made up most of the patients, accounting for 70.4% of the IMN group and 79.6% of the ORIF group. However, compared to the ORIF group (20.4%), the IMN group had a considerably larger percentage of female patients (29.6%) (p=0.03).

The mode of injury showed that high-energy injuries were more common in both groups. In Group A, 41 participants (75.9%) sustained high-energy injuries, while 13 participants (24.1%) had low-energy injuries. In Group B, 45 participants (83.3%) experienced high-energy injuries, and nine participants (16.7%) sustained low-energy injuries. However, the difference between the groups was not statistically significant (p=0.25), indicating that the mode of injury was comparable between both groups.

As regards the distribution of fractures according to the AO/OTA classification between the ORIF and IMN groups, the ORIF group demonstrated a higher proportion of type 33-C (complete intra-articular) fractures (51.9%), indicating a greater number of complex fracture patterns, whereas the IMN group predominantly consisted of type 33-A (extra-articular) fractures (70.4%). This difference in fracture distribution between the two groups was found to be statistically significant (χ²=28.42; p<0.001), suggesting baseline variation in fracture complexity between surgical groups.

To compare the postoperative functional mobility between the groups, knee flexion ROM in patients treated with ORIF and IMN was categorized into four ranges: >125°, 100-124°, 90-99°, and <89°. Compared to the ORIF group (22%), a considerably greater percentage of patients in the IMN group (42%) attained >125° knee flexion. Both groups' results in the 100-124° range were similar, with IMN at 25% and ORIF at 27.7%.

Compared to IMN (20%), ORIF had a larger proportion of patients in the 90-99° range (35%), suggesting that the ORIF group had more moderate restrictions. Severe restrictions (<89°) were more common in ORIF (14%) compared to IMN (11%). The IMN group demonstrated noticeably better outcomes in reaching greater knee flexion as compared to the ORIF group. The statistical significance of the differences in knee flexion between the two groups is confirmed by a T-value of 2.03 and a p-value of 0.04, as given in Table 2.

**Table 2:** Range of motion ORIF: open reduction-internal fixation; IMN: intramedullary nailing; χ²: chi-squared value; t: Student's t-test value; F: ANOVA F-statistic

Knee flexion (range)	ORIF (n=54)	IMN (n=54)	Test statistic	P-value
>125°	12 (22%)	23 (42%)	χ²=8.24	0.041
100-124°	15 (27.7%)	14 (25%)	t=2.03	0.04
90-99°	19 (35%)	11 (20%)	F=3.82	0.053
<89°	8 (14%)	6 (11%)	-	-

Regarding the Manual Muscle Testing findings for the quadriceps and hamstring muscle groups at the one-year follow-up, in the quadriceps muscle group, 55% of patients demonstrated full muscle strength (Grade 5/5), while 96% achieved at least Grade 4/5, indicating satisfactory postoperative recovery in most participants. Similarly, in the hamstring muscle group, 64% of patients achieved Grade 5/5, and 92% demonstrated at least Grade 4/5 strength, as given in Table 3.

**Table 3:** Manual Muscle Testing

	Manual Muscle Testing score	Number of individuals	Percentage
Quadriceps muscle group	5 to 5	30	55%
	4 to 5	52	96%
	3 to 5	28	48%
Hamstring muscle group	5 to 5	35	64%
	4 to 5	50	92%
	3 to 5	23	42%

The most prevalent aberrant pattern was antalgic gait (29.6%). Additionally, common was the knee flexion avoidance gait (20.4%), which may indicate quadriceps weakness or stiffness. Reduced knee flexion in swing (11.1%) was the least prevalent, associated with stiffness or weak muscles, while quadriceps avoidance gait (14.8%) suggested a deficit in the extensor mechanism. Overall, the primary causes of abnormal gait following surgery were pain and stiffness, as given in Table 4.

**Table 4:** Gait pattern

Gait pattern	No. of individuals	%
Antalgic gait	32	29.6%
Knee flexion avoidance gait	22	20.4%
Quadriceps avoidance gait	16	14.8%
Reduced knee flexion in swing	12	11.1%

Complications observed in patients treated with ORIF and IMN were compared between the groups based on knee stiffness, limb shortening, muscle wasting, infection, implant failure, and extensor lag. The findings are shown together with the t-values and p-values for statistical significance that correspond to them. Patients treated with IMN show a significantly better ROM compared to ORIF (t-value=2.03; p=0.04), indicating statistical significance. Most patients in both groups do not exhibit limb shortening, indicating a similar pattern. Leg shortening is not present in the majority of individuals in each group, suggesting a comparable pattern. There was no statistically significant variation in the groups' shortening (t-value=0.883; p=0.829). A high incidence of muscle wasting is observed in both groups. There was no alteration in extensor lag which is statistically significant (t-value=1.43; p=0.155). A slightly higher rate of infection is noted in the ORIF group, but it is not statistically significant (t-value=0.56; p=0.754). Implant failure is rare in both groups, with no significant difference. The IMN group has a higher success rate in achieving a normal ROM as a result of knee stiffness (p=0.04). There were no significant variations in shortening, muscle atrophy, infection, or implant failure between the two groups. Extensor lag is slightly less severe in the IMN group, but the difference is not statistically significant. IMN appears to be the better option for functional recovery (normal ROM and extensor lag), while ORIF shows slightly higher complications in stiffness and infection, as given in Table 5.

**Table 5:** Various complications observed in patients ORIF: open reduction-internal fixation; IMN: intramedullary nailing; χ²: chi-squared value; t: Student's t-test value; F: ANOVA F-statistic

Complication/variable	ORIF (n=54)	IMN (n=54)	t-/χ²-value	F-value	P-value
Knee stiffness (range of motion)					
Normal (>125°)	12 (22%)	23 (42%)	χ²=8.24	F=3.82	0.041
Mild (100-124°)	15 (27.7%)	14 (25%)			
Moderate (90-99°)	19 (35%)	11 (20%)			
Severe (<89°)	8 (14%)	6 (11%)			
Shortening (cm)					
Normal (0)	36 (66%)	38 (70.4%)	t=0.883	F=0.780	0.829
Mild (<1.5)	8 (14.8%)	9 (16.7%)			
Moderate (1.5-2.5)	6 (11%)	5 (9%)			
Severe (>2.5)	4 (7%)	2 (3.7%)			
Extensor lag (degrees)					
Normal (0)	10 (18%)	14 (25%)	t=1.43	F=2.04	0.155
Mild (<5°)	18 (33%)	21 (38%)			
Moderate (6-10°)	14 (25%)	11 (20%)			
Severe (>10°)	12 (22%)	8 (14%)			
Other surgical complications					
Muscle wasting	45 (83%)	41 (75%)	χ²=0.98	-	0.321
Infection	4 (8%)	2 (4%)	t=0.56	-	0.754
Implant failure	3 (6%)	2 (4%)	χ²=0.21	-	0.648

Table 6 demonstrates that individuals who had surgery with IMN had better overall functional outcomes compared to those who had ORIF.

**Table 6:** Functional outcome assessed using the Schatzker and Lambert criteria ORIF: open reduction-internal fixation; IMN: intramedullary nailing

Outcome category	ORIF (n=54)	IMN (n=54)	t-value	χ²-value	F-value (ANOVA)	P-value
Excellent	11 (20.4%)	26 (48.1%)	2.53	10.22	6.40	0.012
Good	19 (35.2%)	13 (24.1%)				
Fair	16 (29.6%)	12 (22.2%)				
Poor	8 (14.8%)	3 (5.6%)				

## Discussion

The present study assessed the postoperative functional impairments in patients surgically treated for distal femur fractures, with particular focus on physiotherapy-related outcomes. Our findings revealed that dysfunction was common and multifactorial, with knee stiffness, muscle wasting, extensor lag, gait deviations, and limb shortening contributing to significant limitations in mobility. These results are consistent with previous literature, which emphasizes that distal femur fractures, though surgically stabilized, frequently lead to long-term morbidity and reduced quality of life. Although less frequent compared to other long-bone injuries, distal femur fractures are known to cause considerable morbidity due to their anatomical complexity and critical role in knee biomechanics [14]. While surgical fixation provides stability and enables early mobilization, long-term functional impairments often persist and require careful physiotherapy assessment. Distal femoral fractures are difficult to treat. Most failures are caused by poor fracture fragment fixation [16].

The characteristics of distal femoral fractures are influenced by factors such as the patient's age, intra-articular extension, degree of comminution, bone quality, treatment technique, implant choice, and timing of physiotherapy. The primary aim of treatment is to achieve a stable, painless joint with a normal or near-normal ROM. This can be accomplished using an implant that provides rigid fixation of articular fragments, preserves surrounding soft tissue, maintains vascular supply, and allows early weight-bearing. In younger individuals, distal femoral fractures often result from high-energy trauma, leading to comminuted or open fractures. In contrast, elderly individuals with osteopenic or osteoporotic bone may sustain these fractures from low-energy mechanisms. Supracondylar femur fractures can be managed using two principal approaches: IMN or ORIF. In our cohort, IMN was associated with better knee flexion outcomes compared to ORIF. Specifically, 42% of patients in the IMN group achieved >125° flexion, compared to 22% in the ORIF group. This finding is clinically meaningful, as greater flexion angles are directly associated with improved performance of daily activities such as squatting, stair climbing, and rising from a seated position. Previous studies have similarly reported that IMN, being less invasive and preserving periosteal blood supply, tends to result in superior ROM outcomes compared to plate fixation [17].

Quadriceps weakness was another consistent finding in our study. Muscle wasting and quadriceps weakness were also frequent findings, particularly in patients with delayed mobilization. Muscle atrophy is an inevitable consequence of both immobilization and disuse following major lower limb trauma [18]. Previous studies observed significant quadriceps wasting in patients after distal femoral fixation, which correlated strongly with extensor lag and reduced walking endurance.** **Physiotherapy evaluation of muscle bulk and strength is therefore critical in understanding long-term outcomes and guiding rehabilitation priorities. While many patients recovered near-normal strength (MMT ≥4/5), less than half regained full (5/5) quadriceps strength. The differences in surgical approach between retrograde IMN and ORIF may also contribute to variations in postoperative function and long-term outcomes. IMN is typically performed using a minimally invasive technique with relatively less soft tissue disruption and preservation of periosteal blood supply, which may facilitate earlier mobilization and functional recovery. In contrast, ORIF with locked plating generally requires open reduction and greater soft tissue exposure, particularly in complex intra-articular fractures, which may increase postoperative pain, joint stiffness, and delayed rehabilitation. These inherent procedural differences should be considered while interpreting postoperative functional outcomes between the two groups.

Comparable findings have been documented in lower limb fracture populations, where compensatory gait mechanics often persist despite fracture healing [18]. Physiotherapy assessment of gait is essential not only for describing deviations but also for identifying their underlying causes, which may vary depending on the surgical method and individual patient factors. Functional outcomes based on the Schatzker and Lambert criteria showed significantly more "excellent" results in the IMN group compared to the ORIF group, underscoring the influence of fixation method on long-term recovery. Importantly, complications such as knee stiffness and mild infection were more common among ORIF patients, which may have hindered mobility and functional progress [17]. From a physiotherapy perspective, such complications highlight the need for ongoing monitoring of joint mobility, early recognition of extensor lag, and structured assessment of activities of daily living to accurately capture their impact on patient independence.

In summary, this study demonstrates that although surgical fixation restores stability in distal femur fractures, substantial postoperative functional limitations remain. Key physiotherapy-related findings included restricted ROM, quadriceps weakness, and persistent gait deviations, with outcomes varying according to the fixation method. These results reinforce the essential role of physiotherapy assessment in postoperative care, providing a framework to identify deficits that extend beyond radiological healing. Future studies should incorporate standardized functional outcome measures and longitudinal physiotherapy evaluations to better capture recovery trajectories and guide evidence-based rehabilitation strategies.

Limitations

This study has several limitations that should be acknowledged. First, the study was conducted at a single center with a relatively limited sample size, which may affect the generalizability of the findings. Second, the cross-sectional study design limits the ability to establish long-term causal relationships regarding postoperative functional outcomes. Third, pre-injury functional status and baseline pain levels were not available, which may have influenced postoperative functional assessment. Fourth, detailed information regarding individual physiotherapy rehabilitation adherence, duration, and intensity was not separately recorded, preventing subgroup analysis based on rehabilitation exposure. Finally, comparison between ORIF and IMN should be interpreted cautiously, as differences in fracture complexity and surgical indications between the two groups may have influenced postoperative outcomes.

Strengths

The present study has several strengths. It provides a comprehensive evaluation of postoperative knee dysfunction following surgically managed distal femur fractures using multiple objective clinical outcome measures, including ROM, Manual Muscle Testing, limb length assessment, girth measurement, gait analysis, and functional outcome assessment using the Schatzker and Lambert criteria. Inclusion of both ORIF and IMN groups allowed the assessment of postoperative functional patterns across different surgical approaches. Additionally, the study highlights clinically relevant postoperative complications and functional impairments, contributing useful evidence to support rehabilitation planning and patient management following distal femur fracture surgery.

## Conclusions

Distal femur fractures, although effectively treated with surgical fixation to achieve anatomical alignment, often lead to persistent postoperative dysfunctions. Our study found that knee stiffness, muscle wasting, extensor lag, limb shortening, and gait abnormalities remain common despite successful surgical union, significantly limiting functional recovery and daily mobility. These findings underscore the importance of early, targeted physiotherapy interventions. Rehabilitation should focus on restoring knee ROM, progressive muscle strengthening, and gait re-education. Additionally, physiotherapy-based assessments offer a more comprehensive evaluation of recovery by capturing the full spectrum of functional deficits. Initiating structured physiotherapy along with patient education soon after surgery can accelerate recovery, reduce postoperative disability, and enhance long-term quality of life. Integrating systematic physiotherapy evaluation into routine follow-up also helps identify patients at risk of prolonged disability and emphasizes the need for individualized rehabilitation strategies.
